# How does re-classification of variants of unknown significance (VUS) impact the management of patients at risk for hereditary breast cancer?

**DOI:** 10.1186/s12920-022-01270-4

**Published:** 2022-05-31

**Authors:** Ava Kwong, Cecilia Yuen Sze Ho, Vivian Yvonne Shin, Chun Hang Au, Tsun-Leung Chan, Edmond Shiu Kwan Ma

**Affiliations:** 1 Chief of Breast Surgery Division, Department of Surgery, The University of Hong Kong and University of Hong Kong-Shenzhen Hospital, Pokfulam, Hong Kong SAR; 2 Department of Surgery, Hong Kong Sanatorium & Hospital, Happy Valley, Hong Kong SAR; 3 Hong Kong Hereditary Breast Cancer Family Registry, Shau Kei Wan, Hong Kong SAR; 4 Department of Molecular Pathology, Hong Kong Sanatorium & Hospital, Happy Valley, Hong Kong SAR

**Keywords:** Hereditary breast cancer, Breast cancer risk, Variants of uncertain significance

## Abstract

**Background:**

The popularity of multigene testing increases the probability of identifying variants of uncertain significance (VUS). While accurate variant interpretation enables clinicians to be better informed of the genetic risk of their patients, currently, there is a lack of consensus management guidelines for clinicians on VUS.

**Methods:**

Among the *BRCA1* and *BRCA2* mutations screening in 3,544 subjects, 236 unique variants (*BRCA1*: 86; *BRCA2*: 150) identified in 459 patients were being reviewed. These variants consist of 231 VUS and 5 likely benign variants at the initial classification.

**Results:**

The variants in 31.8% (146/459) patients were reclassified during the review, which involved 26 unique variants (11.0%). Also, 31 probands (6.8%) and their family members were offered high-risk surveillance and related management after these variants were reclassified to pathogenic or likely pathogenic. At the same time, 69 probands (15%) had their VUS downgraded to cancer risk equivalent to the general population level.

**Conclusion:**

A review of archival variants from *BRCA1* and *BRCA2* genetic testing changed the management for 31.8% of the families due to increased or reduced risk. We encourage regular updates of variant databases, reference to normal population and collaboration between research laboratories on functional studies to define the clinical significances of VUS better.

**Supplementary Information:**

The online version contains supplementary material available at 10.1186/s12920-022-01270-4.

## Introduction

Variations in a genetic sequence are classified as variants of uncertain significance (VUS) when the association with disease risk and the significance to function are unclear. These VUS are usually missense, silent, intronic variants or in-frame deletions and insertions. They have an unknown effect on protein function based on current information. Presently, there is no simple and logical way to determine if these variants are disease-causing or merely represent rare variants associated with no or little cancer risk. With the availability and popularity of next-generation sequencing (NGS), genetic testing has become more common and generally accepted. Sequencing on a single large polymorphic gene, multiple-gene panel, a specific region of the genome or whole exome/genome leads to a rapid expansion of VUS identified. There are insufficient data currently available from functional studies or clinical studies on these large numbers of VUS. This knowledge gap generates significant problems for genetic counselors and clinicians for clinical management in the clinical setting. Clinicians may encounter difficulties interpreting the implications of the genetic test results on cancer risk to their patients and relatives. Appropriate management strategies are crucial to identify patients receiving poly (ADP-ribose) polymerase inhibitors [1] and their relatives, who might require surveillance or prophylactic interventions, and relieve anxiety for those not at increased cancer risk. Unfortunately, the lack of consensus on risk management strategies leads to discrepancies in the counseling of patients and poses difficulties in understanding these "unknown" results [2–4].

There are well-established management guidelines, e.g., those from the National Comprehensive Cancer Network (NCCN) [5] on the pathogenic variants of these two genes. Current management on patients carrying *BRCA* VUS is based on the family’s cancer history to offer breast screening, risk-reducing mastectomy or risk-reducing salpingo-oophorectomy, but no further genetic test on their family according to NCCN [5]. Misinterpretation of VUS might also lead to inaccurate risk perception and biased decisions about prophylactic surgery. We, therefore, reviewed the VUS in high penetrance genes, *BRCA1* and *BRCA2*, which have been studied extensively. Multifactorial analysis has been adopted. Our specific strategy combined the overall likelihood derived from clinical databases, co-occurrence of each VUS with pathogenic variants, personal and family history of both probands and baselines established with normal in-house controls. By understanding the approach to VUS interpretation, appropriate guidelines might be drafted to empower clinicians with plans to harness non-actionable VUS currently from genetic testing to have a better management for these patients and their families.

## Methods

### Ethics statement

The study was performed following the Declaration of Helsinki. Written informed consent was obtained from all participants recruited in this study. This study was approved by the Institutional Review Board of the University of Hong Kong/Hospital Authority West Cluster and respective authorities of other contributing hospitals in Hong Kong.

### Data collection and analysis

A cohort of 3,544 patients with personal cancer or with risk of breast cancers, with or without ovarian cancers, was recruited by the Hong Kong Heredity Breast Cancer Family Registry between 2007 and 2019, who met the selection criteria for genetic testing for hereditary breast and ovarian cancer syndrome [6]. One hundred in-house Chinese female normal controls (mean age: 46.1; range: 20.7–84.9) were included to validate local allelic frequency of single-nucleotide polymorphism (SNP). Genomic DNA from the patient's blood was sequenced on MiSeq or NextSeq (Illumina, San Diego, CA). Paired sequencing reads were mapped to the human reference genome sequence GRCh37/hg19. The references for the *BRCA1* and *BRCA2* genes were NM007294.3 and NM000059.3. Variants from NGS classified/reclassified as pathogenic or likely pathogenic were orthogonally validated by Sanger sequencing.

### Variant interpretation

Called variants with a variant allelic fraction (VAF) of at least 5% were annotated by Ensembl Variant Effect Predictor v75 [7]. Variants with a minor allele frequency of at least 1% reported by The 1000 Genomes Projects [8] and presented in our in-house normal reference control were excluded from manual variant curation. Variants were labeled according to the Human Genome Variation Society (HGVS). Variants in this study were interpreted and assigned classifications recommended by the American College of Medical Genetics and Genomics (ACMG) and the Association for Molecular Pathology (AMP) guidelines [9] into five-tier classes: pathogenic, likely pathogenic, uncertain significance, likely benign, and benign.

### Variants reclassification

In this study, all VUSs and those classified as likely benign identified from 2007–2019 were reviewed annually starting from 2018. Clinical consideration regarding evidence on family studies across ethnicity was considered. We also reviewed evidence derived from the following sources:

#### Reference population, disease-specific, and sequence databases

We reviewed variants with references to Exome Variant Viewer (http://evs.gs.washington.edu/EVS) and Genome Aggregation Database (gnomAD; https://gnomad.broadinstitute.org), which aggregate information from exome or genome sequencing on unselected populations to estimate the frequency of variants in diverse populations. Information was also extracted from other databases, including ClinVar (https://www.ncbi.nlm.nih.gov/clinvar) and the Leiden Open Variation Database LOVD (https://www.lovd.nl). The latter includes combined data from individuals who have personal or family history of the disease or are potential carriers of a predisposition gene. These clinical databases contain information about the variants and variant classifications proposed by the depositor and reviewed by expert panels. VarSome is another database (https://varsome.com) that relies on vast quantities of the databases mentioned above and the in silico analysis prediction programs mentioned below for classifications [10].

Other databases are specific for the *BRCA1* and *BRCA2* genes. BRCA Exchange (https://brcaexchange.org) was an integrated database containing information about pathogenicity, which worldwide data depositors contributed for variants found in *BRCA1* and *BRCA2*. Uniprot (https://www.uniprot.org) is another database that provides a resource of protein sequences and functional information.

#### Segregation analysis

Segregation analysis support confers the penetrance and susceptibility of a variant in a specific disease [11]. Families carrying variants, which were clinically suspicious of pathogenic, were further studied in depth on the genotypes and phenotypes within their pedigree(s). The probabilities for co-segregation of phenotype and genetic variant by chance and the possibility for co-segregation of disease and variants were calculated.

#### Co-Occurrence in trans

Double heterozygosity for *BRCA1* and *BRCA2* mutations is an exceedingly uncommon event [12]. Data suggest that *BRCA1* biallelic pathogenic mutations are embryonically lethal and that homozygosity for *BRCA2* pathogenic mutations results in either lethality or producing a Fanconi anemia phenotype [12]. The co-existence of the variant with a pathogenic mutation in the same gene is potentially interpreted as neutrality.

#### In silico* analysis*

Several prediction tools have emerged as references to classifying these variants. The most commonly used programs are Sorting Intolerant from Tolerant (SIFT, http://sift.jcvi.org/), Polymorphism Phenotyping v2 (PolyPhen-2.1, http://genetics.bwh.harvard.edu/pph2), and Align-Grantham Variation Grantham Deviation (Align-GVGD, http://agvgd.iarc.fr). A recent publication has questioned the reliability of these in silico tools [13], we are still following the current ACMG guidelines, using these aids to assist our analysis.

#### LOH

Patients carrying a germline pathogenic variant in *BRCA* will completely lose the wild-type allele in the tumor [14]. The loss of heterozygosity (LOH) in the tumor samples helps distinguish the rare variant from a deleterious mutation or from a common polymorphism. NGS analysis on the genomic landscapes of somatic tumors from VUS carriers was specifically studied. High mutant allele fraction implied loss of heterozygosity. LOH in tumors implied a strong likelihood that the identified mutation variant should be considered a deleterious mutation rather than a benign polymorphism [15].

#### Reference to the literature on functional studies

Functional assays represent an important tool for assessing the impact of a single amino acid residue change on a specific function or the integrity of a particular protein functional domain. Functional data from mammalian model-based assays to classifying mutation variants would be highly specific and sensitive.

#### Reviewing cancer history of carriers and normal control cohort

Our nursing staff reviewed families on both variant carriers and normal control individuals on the medical records or over the phone for clarification. This study’s last update of reference databases and family studies was in Feb 2021.

## Result

**Fig. 1:**
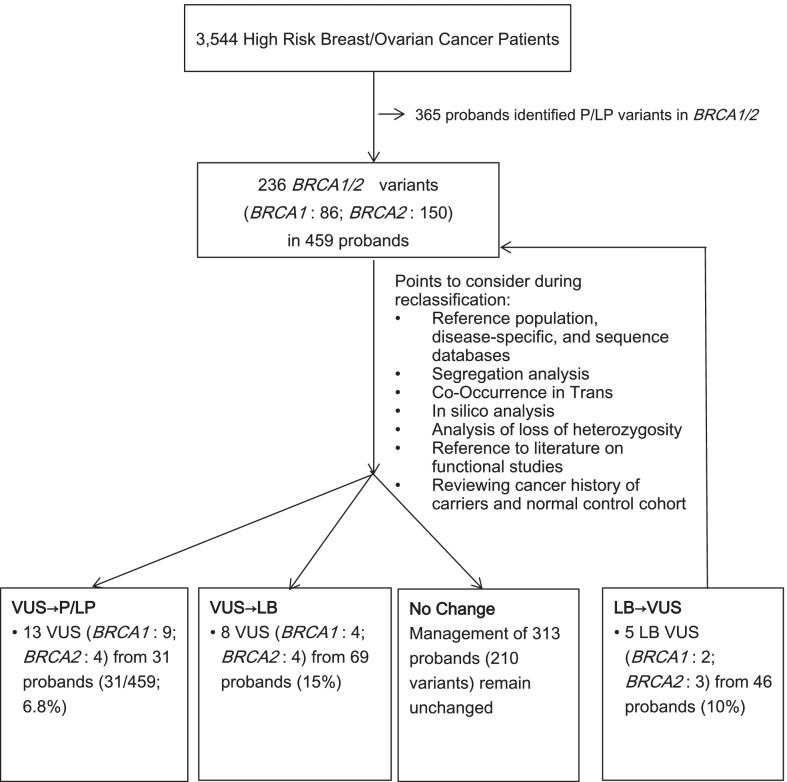
Schematic diagram of BRCA1/2 variants of unknown significance (VUS) identified and reclassified. VUS: variant of uncertain significance; P: Pathogenic; LP: Likely Pathogenic; LB: Likely Benign; B: Benign

This study revealed 3544 breast and ovarian cancer patients who underwent genetic testing for *BRCA* mutations. The rate for pathogenic or likely pathogenic *BRCA* variants was 10.3%. Also, 236 unique VUS and likely benign variants (*BRCA1*: 86; *BRCA2*: 150) were identified in 459 probands, with a rate of 13%. Among these 459 probands, 146 were involved in the reclassification (31.8%). The schematic diagram was shown in Fig. 1. Regular updates of genetic databases and familial studies resulted in the reclassification of 13 VUS to pathogenic or likely pathogenic (*BRCA1*: 9 and *BRCA2*: 4) involving 31 probands (6.8%). The family members of these patients who carried the same variant were offered high-risk surveillance or appropriate risk management according to the National Comprehensive Cancer Network (NCCN) guidelines (Version 3.2021) after reclassification. Moreover, 8 VUS were downgraded to "likely benign" (*BRCA1*: 4 and *BRCA2*: 4). This review resulted in "downgrading" of the initial "suspected" high cancer risk for 69 probands (15%) and their family members who carried the same variant. Furthermore, there were 5 variants (*BRCA1*: 2 and *BRCA2*: 3), involving 46 probands (10%) and their family members, which were originally classified as "likely benign" and were now considered as VUS. Reclassifications were initiated based on the newly emerging cancers in their corresponding normal reference subjects. Distributions and details for the reclassification were listed in Table 1 and Additional files 1 and 2: Table 1a and 1b. The mean and median time differences for the date of the first report to the date of reclassification were 57.5 months and 55.4 months, ranging from 1.3 to 149.4 months. With the adoption of a larger 30 genes panel in our previous study [16], 15 probands that carried uncertain significance (Class 3) *BRCA1* and *BRCA2* variants were found to carry mutations in other cancer predisposition genes with high to moderate penetrance. These mutations were classified as "likely pathogenic" (Class 4) or "pathogenic" (Class 5) (details were shown in Additional file 3: Table 2), and these patients were also offered high-risk surveillance management according to the NCCN guidelines.

**Table 1:** Number of affected families due to reclassified variants

Re-classification	Involved variants	Involved probands
Increased risk (VUS → Pathogenic/Likely pathogenic)	13 (5.5%)	31 (6.8%)
Downgrade of risk (VUS → Likely Benign)	8 (3.4%)	69 (15.0%)
Back to potential risk (Likely Benign → VUS)	5 (2.1%)	46 (10.0%)
Remain no change (VUS → VUS)	210 (89.0%)	313 (68.2%)

## Discussion

VUS are primarily missense and splicing variants. The effect on protein function cannot be directly predicted by the changes in genetic coding and the subsequent changes in the amino acid residues. Functional interpretation of missense and small in-frame insertions/deletions is challenging. The clinical impact of a single amino acid residue alternation or in-frame insertion/deletion of small regions in the biochemical and biophysical properties of the protein is difficult to predict. For example, some missense variants (*BRCA1*: c.4484G > T; p.R1495M; *BRCA2*: c.8023A > G; p.I2675V) cause amino acid alternation. However, their effects on the function may vary due to missense change or the splicing, or both [17]. Variants classified as VUS accounted for approximately 5–6% of mutations found in the single-gene testing [9]. With the recent increase in the use of NGS technology platforms, there were corresponding expansions in the number of genes tested, and a great increase in the number of VUS identified. This trend of increasing VUS identification is especially significant in the less common or the low-penetrance genes. Increased genetic testing in different ethnic groups further broadened the spectrum of mutations and increased the number of reported VUS [18–21]. Genetic tests in African-American ancestry showed that 21% of them carried a VUS in either *BRCA1* or *BRCA2* genes [17]. It was estimated that up to 15% of VUS in *BRCA1* and *BRCA2* were identified among individuals of European descendants [18]. The *BRCA1* and *BRCA2* VUS rates varied from 3.3 to 4.4% across different races, and were highest in Asian origin [19, 20]. The combined *BRCA1* and *BRCA2* VUS rate in a cohort of 21,216 Chinese breast cancer cohort was 9.8% [21]. This study reported that 13.0% of our high-risk cohort had one or more *BRCA1* and *BRCA2* VUS (Fig. 1).

Reclassification of VUS has been emphasized in some recent studies [1, 21, 22]. In this study and other studies, combined various factors was to evaluate the pathogenicity of a variant. These adjustments were based on peer-reviewed literature, multifactorial likelihood models that use personal and family history of cancer, segregation data, in-silico prediction tools, co-occurrence with a pathogenic variant, clinical practice guidelines and large-scale cancer mutation databases [23–25]. A total of 26 variants (1.1%) were being reclassified between 2018–2021, where the majority of the VUS cases were identified before 2018 (84.2%), with reference to data available at that time. Firstly, half of the variants (13 out of 26) were reclassified from recent clinical databases updates. Secondly, we have now collected more families with VUS and more co-segregation studies have been done. With the expansion of our co-segregation studies, 8 out of 26 (30.7%) of our VUS were considered for reclassification. Thirdly, functional assay on 100 variants in *BRCA1* identified in the Chinese population allowed 43.6% of their VUS reclassifying as pathogenic [21]. However, there are significant hurdles with this functional data approach, as functional studies on VUS are limited. Only 3 of our VUS had supporting evidence on the loss of function and were reclassified. We have further confirmed LOH or induced alternative splicing in four of our variants. We actively called back the corresponding patients to retrieve the tissues from different sources and extra blood processing for RNA works. This approach thereby increased the costs of the somatic tests and the administrative workload. Fourthly, two likely benign variants were upgraded to VUS because the clinical and family history of our normal control group was updated. As the information is being updated continuously, the classification of VUS has to be reviewed regularly. Patients and their family members should be informed of these latest findings promptly. We noticed a study suggesting classifying uncharacterized missense variants by identifying cold-spot regions in the *BRCA1* and *BRCA2* genes [22]. In our cohort, seven of our reclassified VUS in our cohort are located in the suggested cold-spot regions, four of these variants were reclassified as likely benign and three were reclassified from likely benign to VUS. The usefulness of this approach of identifying cold-spots within genes for VUS still needs to be evaluated.

Besides the continuous change of references, there is also a significant inter-laboratory discrepancy in the classification of genetic variants due to the differences in statistical thresholds, reference databases and levels of evidence to consider in the prediction models [26, 27]. Some missense variants may have very low frequencies (< 1/10,000) in a particular population. The low frequency would impair the clinical explication of these variants from linkage analysis. The unavailability of family genetic information and cancer history hampered the likelihood analysis and increased the complexity of variant interpretation [28]. Such situation is a challenging scenario for clinicians to assess the pathogenicity of a variant when VUS arises in the families where the epidemiological data are sparse or not available [29]. Another difficult situation is when pieces of evidence are incongruous with each other. These are the variants that are shown with conflicting interpretations of pathogenicity. There are variants that are interpreted between likely pathogenic and variants of unknown significance in these situations. If interpretation of the significance of these variants is not supported by strong evidence, such as functional assay, we tend to keep it as VUS and put these subjects under the dynamic cycle of review. Our rationale is to avoid imposing irreversible decisions on these subjects. In other scenario, where the interpretations of variants conflict between VUS against those of "likely benign", we would favor to classify them as VUS. This practice is based on “patients’ right to information”. Special notes will be supplemented in the report to alert the clinicians on the interpretations of these VUS. It is the purview of the clinician's judgment based on the evidence from different sources [29] and integrate the functional studies from international and inter-laboratory collaboration or sharing of validated data into their clinical practices [30]. Variant classification forms the basis for clinical judgement, including diagnosis, prognosis, therapy selection, and monitoring of therapy. There is a common mistaken belief on the five-tier system, in which the difference between each class reflects the strength of evidence available instead of the levels of risk [21]. The possible reason for this misassumption is that the missense variants which incapacitate protein function will have a moderate risk [31]. A VUS may appear pathogenic in a particular family or multiple families if an unidentified pathogenic variant is *cis* in the genetic region or in other genes that were not covered in the test panel [32]. Special attention is given when assigning pathogenicity to such variants, particularly with racial and ethnic disparities and normal population control. Some variants may appear as single-nucleotide polymorphism (SNP) and are commonly seen in Asians but not in other ethnic groups. Thus, the functional data to support the VUS classification would be crucial. Clinicians can base on the aforementioned consideration to aid medical decision-making on managing risk reduction and surveillance options. Freely available access and accurate information on variants are also important factors in implementing precision medicine. However, as data are being updated continually, routine procedures or automatic programming to review the status of previously reported VUS in literature and databases is necessary; therefore, updated clinical report should be issued as new information. It is crucial to keep accurate patient contact information and revisit patient clinical or family histories, even after the patient has been discharged from the genetics clinic. These can be facilitated by establishing a cancer family registry to review and update the database regularly. In our registry, the mean time difference between the case received and the date of reclassification was 57.5 months, ranged from 1.3 to 149.4 months. The re-issued reports and informed probands and their families to review new information is imperative when a VUS is re-classified as pathogenic or benign. Furthermore, such re-classification reduces patients’ mental stress due to VUS-associated uncertainty.

## Conclusion

The review of archival VUS derived from *BRCA1* and *BRCA2* genetic testing changed the management of many families in the direction of either increased or diminished risk. Multiple-gene testing provides more genetic information to clinicians and patients. Due to the limitation of studies on different genes and rapid progress in translational research, clinicians might be more conservative on medical management decisions based on VUS findings with limited clinical guidelines. We encourage regular updates of variant databases, clinical data from both probands and family members who carried a VUS and normal population reference. Collaboration with research laboratories on functional studies for VUS is encouraged to understand their clinical significance better.

## Supplementary Information

**Additional file 1.** **Supplementary Table 1a.** Reclassified variant of uncertain significance (VUS) with evidences.

**Additional file 2.** **Supplementary Table 1b.** Probands carry reclassified variant of uncertain significance (VUS).

**Additional file 3.** **Supplementary Table 2.** *BRCA1/2* VUS probands carried germline pathogenic/likely pathogenic mutations in genes other than *BRCA1/2*.

**Additional file 4. Supplementary Table 3.** List of reclassified variant of uncertain significance

## Data Availability

The dataset supporting the conclusions of this article is included within the article and its additional files. Mutated sequences have been submitted to Genbank, accession numbers of the sequences were listed in Additional file 4: Table 3 and were released for public access.

## Abbreviations

VUS: Variants of unknown significance
NGS: Next-generation sequencing
NCCN: The National Comprehensive Cancer Network
VAF: Variant allele frequency
HGVS: Human Genome Variation Society
ACMG: American College of Medical Genetics and Genomics
AMP: Association for Molecular Pathology
LOH: Loss of heterozygosity

## References

[1] Lee JS, Oh S, Park SK, Lee MH, Lee JW, Kim SW (2018). Reclassification of *BRCA1* and *BRCA2* variants of uncertain significance: a multifactorial analysis of multicentre prospective cohort. J Med Genet.

[2] Eccles BK, Copson E, Maishman T, Abraham JE, Eccles DM (2015). Understanding of BRCA VUS genetic results by breast cancer specialists. BMC Cancer.

[3] Scherr CL, Lindor NM, Malo TL, Couch FJ, Vadaparampil ST (2015). Genetic counselors’ practices and confidence regarding variant of uncertain significance results and re-classification from BRCA testing. Clin Gen.

[4] Eccles DM, Mitchell G, Monteiro AN, Schmutzler R, Couch FJ, Spurdle AB (2015). BRCA1 and BRCA2 genetic testing-pitfalls and recommendations for managing variants of uncertain clinical significance. Ann Oncol.

[5] NCCN Clinical Practice Guidelines in Oncology (NCCN Guidelines). 2021. Genetic/familial high-risk assessment: Breast and ovarian. https://www2.tri-kobe.org/nccn/guideline/gynecological/english/genetic_familial.pdf. Accessed 15 Jan 2021. 10.6004/jnccn.2021.0001 33406487

[6] Kwong A, Shin VY, Au CH, Law FB, Ho DN, Ip BK (2016). Detection of germline mutation in hereditary breast and/or ovarian cancers by next-generation sequencing on a four-gene panel. J Mol Diagn.

[7] McLaren W, Gil L, Hunt SE, Riat HS, Ritchie GR, Thormann A (2016). The ensembl variant effect predictor. Genome Biol.

[8] Auton A, Brooks LD, Durbin RM, Garrison EP, Kang HM, 1000 Genomes Project Consortium (2015). A global reference for human genetic variation. Nature.

[9] Richards S, Aziz N, Bale S, Bick D, Das S, Gastier-Foster J (2015). Standards and guidelines for the interpretation of sequence variants: a joint consensus recommendation of the American College of Medical Genetics and Genomics and the Association for Molecular Pathology. Genet Med.

[10] Kopanos C, Tsiolkas V, Kouris A, Chapple CE, Albarca Aguilera M, Meyer R (2019). VarSome: the human genomic variant search engine. Bioinformatics.

[11] Møller P, Clark N, Mæhle L (2011). A Simplified method for Segregation Analysis (SISA) to determine penetrance and expression of a genetic variant in a family. Hum Mutat.

[12] Zuradelli M, Peissel B, Manoukian S, Zaffaroni D, Barile M, Pensotti V (2010). Four new cases of double heterozygosity for BRCA1 and BRCA2 gene mutations: clinical, pathological, and family characteristics. Breast Cancer Res Treat.

[13] Gunning AC, Fryer V, Fasham J, Crosby AH, Ellard S, Baple EL, et al. Assessing performance of pathogenicity predictors using clinically relevant variant datasets. *J Med Genet*. 2020;jmedgenet-2020–107003. 10.1136/jmedgenet-2020-107003 PMC8327323 32843488

[14] Osorio A, de la Hoya M, Rodríguez-López R, Martínez-Ramírez A, Cazorla A, Granizo JJ (2002). Loss of heterozygosity analysis at the BRCA loci in tumor samples from patients with familial breast cancer. Int J Cancer.

[15] Sorscher S, Ramkissoon S (2017). Next-Generation Sequencing in Order to Better Characterize a BRCA Variant of Uncertain Significance. Case Rep Oncol.

[16] Kwong A, Shin VY, Chen J, Cheuk IW, Ho CY, Au CH (2020). Germline mutation in 1,338 BRCA-negative Chinese hereditary breast and/or ovarian cancer patients: clinical testing with a multigene test panel. J Mol Diagn.

[17] Ready K, Gutierrez-Barrera AM, Amos C, Meric-Bernstam F, Lu K, Hortobagyi G (2011). Cancer risk management decisions of women with BRCA1 or BRCA2 variants of uncertain significance. Breast J.

[18] Lindor NM, Goldgar DE, Tavtigian SV, Plon SE, Couch FJ (2013). BRCA1/2 sequence variants of uncertain significance: a primer for providers to assist in discussions and in medical management. Oncologist.

[19] Frank TS, Deffenbaugh AM, Reid JE, Hulick M, Ward BE, Lingenfelter B (2002). Clinical characteristics of individuals with germline mutations in BRCA1 and BRCA2: analysis of 10,000 individuals. J Clin Oncol.

[20] Caswell-Jin JL, Gupta T, Hall E, Petrovchich IM, Mills MA, Kingham KE (2018). Racial/ethnic differences in multiple-gene sequencing results for hereditary cancer risk. Genet Med.

[21] Liu Y, Wang H, Wang X, Liu J, Li J, Wang X (2021). Prevalence and reclassification of BRCA1 and BRCA2 variants in a large, unselected Chinese Han breast cancer cohort. J Hematol Oncol.

[22] Dines JN, Shirts BH, Slavin TP, Walsh T, King MC, Fowler DM (2020). Systematic misclassification of missense variants in BRCA1 and BRCA2 "coldspots". Genet Med.

[23] Easton DF, Deffenbaugh AM, Pruss D, Frye C, Wenstrup RJ, Allen-Brady K (2007). A systematic genetic assessment of 1,433 sequence variants of unknown clinical significance in the BRCA1 and BRCA2 breast cancer-predisposition genes. Am J Hum Genet.

[24] Goldgar DE, Easton DF, Deffenbaugh AM, Monteiro AN, Tavtigian SV, Couch FJ (2004). Integrated evaluation of DNA sequence variants of unknown clinical significance: application to BRCA1 and BRCA2. Am J Hum Genet.

[25] Lindor NM, Guidugli L, Wang X, Vallée MP, Monteiro AN, Tavtigian S (2012). A review of a multifactorial probability-based model for classification of BRCA1 and BRCA2 variants of uncertain significance (VUS). Hum Mutat.

[26] Eggington JM, Bowles KR, Moyes K, Manley S, Esterling L, Sizemore S (2014). A comprehensive laboratory-based program for classification of variants of uncertain significance in hereditary cancer genes. Clin Genet.

[27] Pesaran T, Karam R, Huether R, Li S, Farber-Katz S, Chamberlin A (2016). Beyond DNA: an integrated and functional approach for classifying germline variants in breast cancer genes. Int J Breast Cancer.

[28] Golubeva VA, Nepomuceno TC, Monteiro ANA (2019). Germline missense variants in *BRCA1*: new trends and challenges for clinical annotation. Cancers (Basel).

[29] Eccles BK, Copson E, Maishman T (2015). Understanding of BRCA VUS genetic results by breast cancer specialists. BMC Cancer.

[30] Li MM, Datto M, Duncavage EJ, Kulkarni S, Lindeman NI, Roy S (2017). Standards and guidelines for the interpretation and reporting of sequence variants in cancer: a joint consensus recommendation of the Association for Molecular Pathology, American Society of Clinical Oncology, and College of American Pathologists. J Mol Diagn.

[31] Lyra PCM, Nepomuceno TC, de Souza MLM, Machado GF, Veloso MF, Henriques TB (2021). Integration of functional assay data results provides strong evidence for classification of hundreds of BRCA1 variants of uncertain significance. Genet Med.

[32] Lovelock PK, Spurdle AB, Mok MT, Farrugia DJ, Lakhani SR, Healey S (2007). Identification of BRCA1 missense substitutions that confer partial functional activity: potential moderate risk variants?. Breast Cancer Res.

[33] Nelson AC, Holt JT (2010). Impact of RING and BRCT domain mutations on BRCA1 protein stability, localization and recruitment to DNA damage. Radiat Res.

[34] Findlay GM, Daza RM, Martin B, Zhang MD, Leith AP, Gasperini M (2018). Accurate classification of BRCA1 variants with saturation genome editing. Nature.

[35] Lee MS, Green R, Marsillac SM, Coquelle N, Williams RS, Yeung T (2010). Comprehensive analysis of missense variations in the BRCT domain of BRCA1 by structural and functional assays. Cancer Res.

[36] Petitalot A, Dardillac E, Jacquet E, Nhiri N, Guirouilh-Barbat J, Julien P (2019). Combining homologous recombination and phosphopeptide-binding data to predict the impact of *BRCA1* BRCT variants on cancer risk. Mol Cancer Res.

[37] Mesman RLS, Calléja FMGR, Hendriks G, Morolli B, Misovic B, Devilee P (2019). The functional impact of variants of uncertain significance in BRCA2. Genet Med.

[38] Richardson ME, Hu C, Lee KY, LaDuca H, Fulk K, Durda KM (2021). Strong functional data for pathogenicity or neutrality classify BRCA2 DNA-binding-domain variants of uncertain significance. Am J Hum Genet.

