# Supplementary material for: How does re-classification of variants of unknown significance (VUS) impact the management of patients at risk for hereditary breast cancer?
Source: BMC Med Genomics. 2022 May 31;15:122. doi: 10.1186/s12920-022-01270-4 (PMC9158111; doi:10.1186/s12920-022-01270-4)
Supplement: Supplementary file 1 — Additional file 1. Supplementary Table 1a. Reclassified variant of uncertain significance (VUS) with evidences. [file 12920_2022_1270_MOESM1_ESM.docx]

Supplementary Table 1a. Reclassified variant of uncertain significance (VUS) with evidences.

| **Gene** | **Mutation Variants** | **Reclassification** | **Mean time* (Month)** | **No. of Probands involved** | **NC^** | **1000 Genome** | **gnomAD** | **ClinVar**  **(classification/ last update date)** | **SIFT** | **PolyPhen** | **Considerations during reclassification** |
| --- | --- | --- | --- | --- | --- | --- | --- | --- | --- | --- | --- |
| *BRCA1* | c.53T>C;  p.Met18Thr | VUS>  Pathogenic | 134.8 | 1 | - | - | - | Pathogenic  19/5/2020 | Deleterious | Benign | - Recent update from ClinVar |
|  | c.116G>A;  p.Cys39Tyr | VUS>  Pathogenic | 104.8 | 1 | - | - | EA: 0/1554  G: 1/31396 | Pathogenic​  18/5/2020 | Deleterious | Probably  damaging | - Recent update from ClinVar |
|  | c.183T>G;  p.Cys61Trp | VUS>  Pathogenic | 56.9 | 1 | - | - | - | absent | Deleterious | Probably damaging | - missense mutation on BRCA1 RING-type Zinc finger domain [33], which has been reported as conserved region in mammal - other non-conservative missense variants (p.Cys61Tyr; p.Cys61Arg; p.Cys61Ser; p.Cys61Gly) with 55 submissions, affecting the same residue have been reported as P/LP in Clinvar. - The saturation genome editing (SGE) assay produced a function score of -2.51, corresponding to “Loss of function” [34] |
|  | c.427G>A;  p.Glu143Lys | VUS>  Likely Benign | 36.5 | 6 | - | - | EA: 8/19922  G: 22/282124 | Benign  24/9/2019 | Tolerated | Benign | - co-seg with *BRCA1* c.1881_1884delCAGT in 2 cases - Recent update from ClinVar |
|  | c.442_444delCAG; p.Gln148del | VUS>  Likely Benign | 149.4 | 1 | - | + | - | Uncertain significance​ 31/1/2014 | No data available for this variant | No data available for this variant | - Common splice site - Coexist with *PALB2* c.1914dupT; p.Glu639* - This variant has been presented in 1000 genome |
|  | c.1033G>T;  p.Asp345Tyr | VUS>  Likely Benign | 12.3 | 7 | - | - | - | Uncertain significance  28/3/2019 | Deleterious | Possibly damaging | - co-seg with BRCA1 c.2635G>T; p.Glu879* in 6 cases |
|  | c.1036C>T;  p.Pro346Ser | VUS>  Likely Benign | 53.1 | 10 | - | - | EA: 54/19952  G: 54/282636 | Benign  18/6/2019 | Tolerated | Benign | - Recent update from ClinVar |
|  | c.2347A>G;  p.Ile783Val | Likely  Benign>  VUS | 24.6 | 12 | - | - | EA: 18/18394  G: 18/250738 | Conflicting interpretations of pathogenicity  26/2/2020 | Tolerated | Benign | - This variant original presented in local normal control - Re-classification due to family hx of brain and CNS tumor in local normal control |
|  | c.3662A>C; p.Glu1221Ala | Likely  Benign>  VUS | 28.1 | 2 | - | - | EA: 7/18388  G: 7/2151296 | Uncertain significance  29/1/2019 | Deleterious | Possibly damaging | - This variant original presented in local normal control - In silico predicted pathogenic - Recent update from ClinVar as VUS |
|  | c.5072C>A; p.Thr1691Lys | VUS>  Likely Pathogenic | 21.6 | 5 | - | - | - | Likely Pathogenic 28/3/2019 | Deleterious | Probably damaging | - Recent update from ClinVar |
|  | c.5089T>C; p.Cys1697Arg | VUS>  Pathogenic | 40.8 | 10 | - | - | - | Pathogenic 30/7/2018 | Deleterious | Probably damaging | - Recent update from ClinVar |
|  | c.5254G>C; p.Ala1752Pro | VUS>  Likely Pathogenic | 55.8 | 1 | - | - | - | Like Pathogenic 30/7/2019 | Deleterious | Benign | - Recent update from ClinVar |
|  | c.5282T>C; p.Phe1761Ser | VUS>  Likely Pathogenic | 119.8 | 1 | - | - | - | Conflicting interpretations of pathogenicity  20/11/2019 | Deleterious | Probably damaging | - 3 submissions in ClinVar of Likely Pathogenic - Functional study shows strong functional effect on BRCA1 BRCT domain [35] and defective HR [36] |
|  | c.5511G>C; p.Trp1837Cys | VUS>  Likely Pathogenic | 105.7 | 2 | - | - | - | Conflicting interpretations of pathogenicity  1/5/2020 | Deleterious | Probably damaging | - Recent update from ClinVar on c.5511G>T; p.Trp1837Cys as Pathogenic, resulting in same amino acid change - Genetic test on tissue showed evidence of LOH (Data not shown) |
|  | c.5521A>C; p.Ser1841Arg | VUS>  Likely  Pathogenic | 71.2 | 3 | - | + | - | Likely pathogenic​ 12/11/2018 | Deleterious | Probably damaging | - Genetic test on tissue showed evidence of LOH (Data not shown) |
| *BRCA2* | c.476-3C>A | VUS>  Pathogenic | 54.9 | 1 | - | - | EA: 0/19952  G: 12/282210 | Uncertain significance​ 14/5/2019 | No data available for this variant | No data available for this variant | - RNA study showed alternative splice variant (Data not shown) |
|  | c.1568A>G;  p.His523Arg | Likely  Benign>  VUS | 33.1 | 19 | - | - | EA: 33/19650  G: 35/277194 | Conflicting interpretations of pathogenicity 26/2/2020 | Tolerated | Benign | - This variant original presented in local normal control - Re-classification due to new development of cancer in local normal control |
|  | c.2350A>G; p.Met784Val | VUS>  Likely  Benign | 1.3 | 1 | - | + | EA: 54/18392  G: 57/250828 | Benign 22/10/2019 | Tolerated | Benign | - Recent update from ClinVar |
|  | c.6325G>A;  p.Val2109Ile | VUS>  Likely Benign | 21.6 | 9 | - | - | EA: 62/19640  G: 62/276480 | Conflicting interpretations of pathogenicity 6/11/2018 | Tolerated | Benign | - Coexist with *BRCA1* c.220C>T; p.Gln74* - Coexist with *TP53* c.818G>A; p.Arg273His - BIC also reported 2carriers who also co-seg with pathogenic variant (*BRCA1* c.4163_4164insA; *BRCA2* c.9076C>T) - This variant was found in 62/19640 control alleles, predominantly observed in the East Asian - This variant is located at a poorly conserved position in mammal |
|  | c.7052C>G; p.Ala2351Gly | VUS>  Likely Benign | 58.4 | 21 | - | - | EA: 40/19952  G: 41/282184 | Conflicting interpretations of pathogenicity 27/12/2017 | Tolerated | Possibly damaging | - Coexist with *BRCA1* c.4372C>T; p.Gln1458* - Coexist with *BRCA1* c.3756_3759delGTCT; p.Ser1253Argfs*10 - BIC also reported 1 carrier who also co-seg with *BRCA2* pathogenic variant (c.1103C>A; p.Ser368Ter) - This variant was found in 40/19952 control alleles, predominantly observed in the East Asian - This variant is located at a poorly conserved position in mammal |
|  | c.7102T>G; p.Leu2368Val | VUS>  Likely Benign | 59.5 | 14 | - | - | EA: 22/19948  G: 22/282366 | Conflicting interpretations of pathogenicity 26/2/2018 | Tolerated | Probably damaging | - co-seg with *BRCA2* c.7878G>A; p.Trp2626* in 4 cases - Coexist with *TP53* c.96+1G>T; r.75_96del; p.Leu26Profs*11 in 1 of our case - This variant was found in 22/19948 control alleles, predominantly observed in the East Asian |
|  | c.7426_7427delinsCC; p.Glu2476Pro | Likely  Benign>  VUS | 25.6 | 3 | + | - | - | Uncertain significance  6/3/2020 | Tolerated | Probably damaging | - This variant original presented in local normal control but absented in gnomAD - Recent update from ClinVar as VUS - Seen in 3 high risk cases |
|  | c.8009C>T; p.Ser2670Leu | VUS>  Likely  Pathogenic | 57.5 | 3 | - | - | - | Conflicting interpretations of pathogenicity 6/3/2020 | Deleterious | Probably damaging | - 7 submissions in ClinVar of Pathogenic/Likely Pathogenic - Genetic test has done on tissue showed evidence of LOH (Data not shown) |
|  | c.8023A>G; p.Met2676_Ile2778del | VUS>  Pathogenic | 63.5 | 1 | - | - | - | Pathogenic​ 18/6/2019 | Deleterious | Possibly damaging | - RNA study showed alternative splice variant of exon 18 (Data not shown) - Recent update from ClinVar |
|  | c.8162T>A; p.Leu2721His | VUS→  Likely  Pathogenic | 71.7 | 1 | - | - | - | Conflicting interpretations of pathogenicity​  17/12/2020 | Deleterious | Probably damaging | - Failed to rescue the cell lethality imposed by Cre-mediated loss of Brca2 [37] - HDR assay showed non-functional [38] |
|  | c.9538C>T; p.Leu3180Phe | Likely  Benign>  VUS | 33.2 | 10 | - | + | EA: 9/18394  G: 10/251368 | Uncertain significance 29/1/2019 | Deleterious | Probably damaging | - presence in 1000 genome - co-seg with *BRCA2*: c.7007G>T; r.6938_7007del70; p.Gly2313Alafs*31 - Seen in 10 high risk cases - 9 cases have done 30 genes extended panel, no other pathogenic event identified - All 8 submissions in ClinVar are as VUS |

*Mean time different between variant re-classification and date of issuing the first mutation variant report

^NC: 100 normal control individuals from local population
