# Supplementary material for: How does re-classification of variants of unknown significance (VUS) impact the management of patients at risk for hereditary breast cancer?
Source: BMC Med Genomics. 2022 May 31;15:122. doi: 10.1186/s12920-022-01270-4 (PMC9158111; doi:10.1186/s12920-022-01270-4)
Supplement: Supplementary file 2 — Additional file 2. Supplementary Table 1b. Probands carry reclassified variant of uncertain significance (VUS). [file 12920_2022_1270_MOESM2_ESM.docx]

Supplementary Table 1b. Probands carry reclassified variant of uncertain significance (VUS).

| **Gene** | **Mutation Variants** | **Reclassification** | **No. of Proband(s)** | **Gender** | **Dx Age** | **Cancer(s)** |
| --- | --- | --- | --- | --- | --- | --- |
| *BRCA1* | c.53T>C; p.Met18Thr | VUS>Pathogenic | 1 | F | 39 | Breast Cancer |
|  | c.116G>A; p.Cys39Tyr | VUS>Pathogenic | 1 | F | 36 | Breast Cancer |
|  | c.183T>G; p.Cys61Trp | VUS>Pathogenic | 1 | F | 30 | Breast Cancer |
|  | c.427G>A; p.Glu143Lys | VUS>Likely Benign | 6 | F | 44 | Breast Cancer |
|  |  |  |  | F | 34 | Breast Cancer |
|  |  |  |  | F | 31 34 | Breast Cancer Breast Cancer |
|  |  |  |  | F | 78 | Peritoneal |
|  |  |  |  | F | 46 | Breast Cancer |
|  |  |  |  | F | 33 | Breast Cancer |
|  | c.442_444delCAG; p.Gln148del | VUS>Likely Benign | 1 | F | 39 63 | Breast Cancer Breast Cancer |
|  | c.1033G>T; p.Asp345Tyr | VUS>Likely Benign | 7 | F | 37 | Breast Cancer |
|  |  |  |  | F | 26 | Breast Cancer |
|  |  |  |  | F | 57 | Breast Cancer |
|  |  |  |  | F | 26 | Breast Cancer |
|  |  |  |  | F | 44 | Ovarian Cancer |
|  |  |  |  | F | 53 | Ovarian Cancer |
|  |  |  |  | F | 57 | Ovarian Cancer |
|  | c.1036C>T; p.Pro346Ser | VUS>Likely Benign | 10 | F | 47 | Breast Cancer |
|  |  |  |  | F | 48 | Breast Cancer |
|  |  |  |  | F | 45 | Breast Cancer |
|  |  |  |  | F | 44 52 | Ovarian Cancer Breast Cancer |
|  |  |  |  | F | 52 | Breast Cancer |
|  |  |  |  | F | 30 | Breast Cancer |
|  |  |  |  | F | 40 | Breast Cancer |
|  |  |  |  | F | 55 | Breast Cancer |
|  |  |  |  | F | 55 | Breast Cancer |
|  |  |  |  | F | 70 70 | Breast Cancer Breast Cancer |
|  | c.2347A>G; p.Ile783Val | Benign>VUS | 12 | F | 84 84 | Breast Cancer Breast Cancer |
|  |  |  |  | M | 56 | Prostate Cancer |
|  |  |  |  | F | 38 | Breast Cancer |
|  |  |  |  | M | 64 | Male Breast Cancer |
|  |  |  |  | F | 42 49 | Thyroid Cancer Breast Cancer |
|  |  |  |  | F | 59 | Ovarian Cancer |
|  |  |  |  | F | 39 | Breast Cancer |
|  |  |  |  | F | 55 | Breast Cancer |
|  |  |  |  | F | 64 64 | Breast Cancer Breast Cancer |
|  |  |  |  | F | 35 | Breast Cancer |
|  |  |  |  | F | 49 | Breast Cancer |
|  |  |  |  | F | NA | No personal cancer |
|  | c.3662A>C; p.Glu1221Ala | Benign>VUS | 2 | F | 52 | Breast Cancer |
|  |  |  |  | F | 39 | Breast Cancer |
|  | c.5072C>A; p.Thr1691Lys | VUS>Likely Pathogenic | 5 | F | 48 62 | Breast Cancer Breast Cancer |
|  |  |  |  | F | 17 | Ovarian Cancer |
|  |  |  |  | F | 37 | Breast Cancer |
|  |  |  |  | F | 57 65 | Ovarian Cancer Breast Cancer |
|  |  |  |  | F | 43 48 54 | Breast Cancer Breast Cancer Peritoneal |
|  | c.5089T>C; p.Cys1697Arg | VUS>Pathogenic | 10 | F | 36 42 | Breast Cancer Breast Cancer |
|  |  |  |  | F | 33 44 46 | Breast Cancer Breast Cancer Ovarian Cancer |
|  |  |  |  | F | 71 | Breast Cancer |
|  |  |  |  | F | 35 37 | Breast Cancer Breast Cancer |
|  |  |  |  | F | 28 36 | Breast Cancer Breast Cancer |
|  |  |  |  | F | 45 | Peritoneal |
|  |  |  |  | F | 46 | Breast Cancer |
|  |  |  |  | F | 52 | Ovarian Cancer |
|  |  |  |  | F | 33 | Breast Cancer |
|  |  |  |  | F | 30 39 | Breast Cancer Breast Cancer |
|  | c.5254G>C; p.Ala1752Pro | VUS>Likely Pathogenic | 1 | F | 27 51 | Breast Cancer Fallopian Tube Cancer |
|  | c.5282T>C; p.Phe1761Ser | VUS>Likely Pathogenic | 1 | F | 30 | Breast Cancer |
|  | c.5511G>C; p.Trp1837Cys | VUS>Likely Pathogenic | 2 | F | 38 38 | Breast Cancer Breast Cancer |
|  |  |  |  | F | 46 50 | Breast Cancer Ovarian Cancer |
|  | c.5521A>C; p.Ser1841Arg | VUS>Likely Pathogenic | 3 | F | 35 36 41 | Breast Cancer Breast Cancer Skin Cancer |
|  |  |  |  | F | 43 51 62 | Breast Cancer Breast Cancer Ovarian Cancer |
|  |  |  |  | F | 51 | Ovarian Cancer |
| *BRCA2* | c.476-3C>A | VUS>Pathogenic | 1 | F | 51 | Breast Cancer |
|  | c.1568A>G; p.His523Arg | Benign>VUS | 19 | F | 38 | Breast Cancer |
|  |  |  |  | F | 46 | Breast Cancer |
|  |  |  |  | F | 67 67 | Breast Cancer Breast Cancer |
|  |  |  |  | F | 43 | Ovarian Cancer |
|  |  |  |  | F | 43 | Breast Cancer |
|  |  |  |  | F | 43 | Breast Cancer |
|  |  |  |  | F | 51 | Breast Cancer |
|  |  |  |  | F | 36 | Breast Cancer |
|  |  |  |  | F | 18 32 | Breast Cancer Breast Cancer |
|  |  |  |  | M | 16 | Acute Myeloid Leukemia |
|  |  |  |  | F | 40 42 | Breast Cancer Breast Cancer |
|  |  |  |  | F | 39 | Breast Cancer |
|  |  |  |  | F | 40 | Breast Cancer |
|  |  |  |  | F | 36 40 | Breast Cancer Uterus Cancer |
|  |  |  |  | F | 42 | Breast Cancer |
|  |  |  |  | F | 61 | Unknown Cancer |
|  |  |  |  | F | 36 | Breast Cancer |
|  |  |  |  | F | 55 55 | Breast Cancer Breast Cancer |
|  |  |  |  | F | 39 | Breast Cancer |
|  | c.2350A>G; p.Met784Val | VUS>Likely Benign | 1 | F | 46 | Breast Cancer |
|  | c.6325G>A; p.Val2109Ile | VUS>Likely Benign | 9 | F | 37 46 | Breast Cancer Breast Cancer |
|  |  |  |  | F | 38 | Breast Cancer |
|  |  |  |  | F | 25 44 | Breast Cancer Breast Cancer |
|  |  |  |  | F | 54 | Breast Cancer |
|  |  |  |  | F | 58 | Breast Cancer |
|  |  |  |  | F | 52 | Breast Cancer |
|  |  |  |  | F | 59 70 | Breast Cancer Breast Cancer |
|  |  |  |  | F | 28 30 | Breast Cancer Breast Cancer |
|  |  |  |  | F | 54 | Ovarian Cancer |
|  | c.7052C>G; p.Ala2351Gly | VUS>Likely Benign | 21 | F | 53 | Breast Cancer |
|  |  |  |  | F | 37 | Breast Cancer |
|  |  |  |  | F | 54 | Breast Cancer |
|  |  |  |  | F | 43 | Breast Cancer |
|  |  |  |  | F | 40 67 68 | Breast Cancer Colorectal Cancers Breast Cancer |
|  |  |  |  | F | 36 | Breast Cancer |
|  |  |  |  | F | 50 50 | Breast Cancer Breast Cancer |
|  |  |  |  | F | 71 | Breast Cancer |
|  |  |  |  | F | 48 | Breast Cancer |
|  |  |  |  | F | 40 | Breast Cancer |
|  |  |  |  | F | 56 | Breast Cancer |
|  |  |  |  | F | 31 33 | Breast Cancer Breast Cancer |
|  |  |  |  | F | 41 41 | Ovarian Cancer Uterus Cancer |
|  |  |  |  | F | 41 | Breast Cancer |
|  |  |  |  | F | 32 | Breast Cancer |
|  |  |  |  | F | 34 | Breast Cancer |
|  |  |  |  | F | 38 | Breast Cancer |
|  |  |  |  | F | 43 | Breast Cancer |
|  |  |  |  | F | 26 50 | Breast Cancer Ovarian Cancer |
|  |  |  |  | F | 41 | Breast Cancer |
|  |  |  |  | F | 39 | Breast Cancer |
|  | c.7102T>G; p.Leu2368Val | VUS>Likely Benign | 14 | F | 32 48 | Breast Cancer Breast Cancer |
|  |  |  |  | F | 48 | Breast Cancer |
|  |  |  |  | F | 41 | Breast Cancer |
|  |  |  |  | F | 18 43 | Breast Cancer Breast Cancer |
|  |  |  |  | F | 45 | Breast Cancer |
|  |  |  |  | F | 70 80 | Breast Cancer Breast Cancer |
|  |  |  |  | F | 42 | Breast Cancer |
|  |  |  |  | F | 34 | Breast Cancer |
|  |  |  |  | F | 55 | Unknown Cancer |
|  |  |  |  | F | 46 | Breast Cancer |
|  |  |  |  | F | 47 | Ovarian Cancer |
|  |  |  |  | F | 55 59 | Breast Cancer Breast Cancer |
|  |  |  |  | F | 39 | Breast Cancer |
|  |  |  |  | F | 31 | Breast Cancer |
|  | c.7426_7427delinsCC; p.Glu2476Pro | Benign>VUS | 3 | F | 46 46 | Ovarian Cancer Uterus Cancer |
|  |  |  |  | F | 71 | Ovarian Cancer |
|  |  |  |  | F | 20 | Breast Cancer |
|  | c.8009C>T; p.Ser2670Leu | VUS>Likely Pathogenic | 3 | F | 41 42 | Breast Cancer Breast Cancer |
|  |  |  |  | F | 48 | Breast Cancer |
|  |  |  |  | F | 51 | Ovarian Cancer |
|  | c.8023A>G; p.Met2676_Ile2778del | VUS>Pathogenic | 1 | F | 40 40 | Breast Cancer Colorectal Cancers |
|  | c.8162T>A;  p.Leu2721His | VUS>Likely Pathogenic | 1 | M | 64 | Male Breast Cancer |
|  | c.9538C>T; p.Leu3180Phe | Benign>VUS | 10 | F | 25 | Breast Cancer |
|  |  |  |  | F | 41 | Breast Cancer |
|  |  |  |  | F | 55 | Breast Cancer |
|  |  |  |  | F | 50 50 UK | Breast Cancer Breast Cancer Cervical Cancer |
|  |  |  |  | M | 47 | Breast Cancer |
|  |  |  |  | F | 52 52 63 | Breast Cancer Thyroid Cancer Breast Cancer |
|  |  |  |  | F | 70 70 | Breast Cancer Breast Cancer |
|  |  |  |  | F | 40 40 | Breast Cancer Breast Cancer |
|  |  |  |  | F | NA | No personal cancer |
|  |  |  |  | F | NA | No personal cancer |
