# Supplementary material for: How does re-classification of variants of unknown significance (VUS) impact the management of patients at risk for hereditary breast cancer?
Source: BMC Med Genomics. 2022 May 31;15:122. doi: 10.1186/s12920-022-01270-4 (PMC9158111; doi:10.1186/s12920-022-01270-4)
Supplement: Supplementary file 3 — Additional file 3. Supplementary Table 2. BRCA1/2 VUS probands carried germline pathogenic/likely pathogenic mutations in genes other than BRCA1/2. [file 12920_2022_1270_MOESM3_ESM.docx]

Supplementary Table 2. *BRCA1/2* VUS probands carried germline pathogenic/likely pathogenic mutations in genes other than *BRCA1/2*.

| **Family ID** | **Personal Cancer History** | **Class 4/5 Mutated Gene Variant** | ***BRCA* Class 3 VUS** | **Classification of VUS** |
| --- | --- | --- | --- | --- |
| T667 | Breast: Dx 55 | *ATM*: c.1262C>G; p.Ser421* | *BRCA2*: c.796T>C; p.Phe266Leu | VUS |
| T1032 | Breast: Dx 27 | *BRIP1*: c.2392C>T; p.Arg798* | *BRCA2*: c.5692G>T; p.Asp1898Tyr | VUS |
| O22 | Ovarian: Dx 42 Colorectal: Dx 42 Corpus uteri: Dx 42 | *MSH2*: c.1457_1460delATGA; p.Asn486Thrfs*10 | *BRCA2*: c.4599A>C; p.Lys1533Asn | VUS |
| T366 | Breast: Dx 32 | *PMS2*: c.1A>G; p.Met1? | *BRCA1*: c.2933A>G; p.Tyr978Cys | VUS |
| T559 | Breast: Dx 43 | *RAD51C*: c.394dupA; p.Thr132Asnfs*23 | *BRCA2*: c.2405A>G; p.Asn802Ser | VUS |
| T370 | Bilateral Breast: Dx 18, 43 | *TP53*: c.96+1G>T; r.75_96del; p.Leu26Profs*11 | *BRCA2*: c.7102T>G; p.Leu2368Val | VUS→Benign |
| O162 | Ovarian: Dx 59 | *RAD51D*: c.270_271dupTA; p.Lys91Ilefs*13 | *BRCA1*: c.2347A>G; p.Ile783Val | Benign→VUS |
| O152 | Ovarian: Dx 43 | *RAD51D*: c.556C>T; p.Arg186* | BRCA2: c.2744C>G; p.Thr915Ser | VUS |
| H22 | Bilateral Breast: Dx 39, 63 | *PALB2*: c.1914dupT; p.Glu639* | *BRCA1*: c.442_444delCAG; p.Gln148del | Benign→VUS |
| H174 | Breast: Dx 24 | *PALB2*: c.1038delA; p.Glu347Asnfs*9 | *BRCA1*: c.235T>C; p.Phe79Leu; *BRCA2*: c.1871C>T; p.Ala624Val | VUS |
| T1423 | Breast: Dx 29 | *RAD51D*: c.270_271dupTA; p.Lys91Ilefs*13 | *BRCA2*: c.2239G>A; p.Glu747Lys | VUS |
| T1685 | Bilateral Breast: Dx 46, 51 | *PALB2*: c.2594C>G; p.Ser865* | *BRCA2*: c.3010A>G; p.Ser1004Gly | VUS |
| T1831 | Breast: Dx 46 | *PALB2*: c.1446delC; p.Ser483Hisfs*2 | *BRCA1*: c.2286A>T; p.Arg762Ser | VUS |
| O430 | Ovarian: Dx 44 | *RAD51C*: c.1000_1003delinsTTTCC; p.Glu334Phefs*14 | *BRCA1*: c.5068A>C; p.Lys1690Gln | VUS |
| T2044 | Breast: Dx 69 | *PALB2*: c.211+1G>A | *BRCA1*: c.2387C>T; p.Thr796Ile | VUS |
