# Supplementary material for: How does re-classification of variants of unknown significance (VUS) impact the management of patients at risk for hereditary breast cancer?
Source: BMC Med Genomics. 2022 May 31;15:122. doi: 10.1186/s12920-022-01270-4 (PMC9158111; doi:10.1186/s12920-022-01270-4)
Supplement: Supplementary file 4 — Additional file 4. Supplementary Table 3. List of reclassified variant of uncertain significance [file 12920_2022_1270_MOESM4_ESM.docx]

**Supplementary Table 3. List of reclassified variant of uncertain significance**

| **Gene** | **Mutation variants** | **GenBank accession numbers** |
| --- | --- | --- |
| *BRCA1* | c.53T>C; p.Met18Thr | OK018154 |
| *BRCA1* | c.116G>A; p.Cys39Tyr | MG494352 |
| *BRCA1* | c.183T>G; p.Cys61Trp | MG494356 |
| *BRCA1* | c.427G>A; p.Glu143Lys | OK018160 |
| *BRCA1* | c.442_444delCAG; p.Gln148del | OK018161 |
| *BRCA1* | c.1033G>T; p.Asp345Tyr | OK018162 |
| *BRCA1* | c.1036C>T; p.Pro346Ser | OK018163 |
| *BRCA1* | c.2347A>G; p.Ile783Val | OK018164 |
| *BRCA1* | c.3662A>C; p.Glu1221Ala | OK018165 |
| *BRCA1* | c.5072C>A; p.Thr1691Lys | MZ032021 |
| *BRCA1* | c.5089T>C; p.Cys1697Arg | OK018155 |
| *BRCA1* | c.5254G>C; p.Ala1752Pro | OK018157 |
| *BRCA1* | c.5282T>C; p.Phe1761Ser | OK018158 |
| *BRCA1* | c.5511G>C; p.Trp1837Cys | OK018156 |
| *BRCA1* | c.5521A>C; p.Ser1841Arg | OK018159 |
| *BRCA2* | c.476-3C>A; r.426_516del; p.Ser142Argfs*13 | MZ803008 |
| *BRCA2* | c.1568A>G; p.His523Arg | OK018166 |
| *BRCA2* | c.2350A>G; p.Met784Val | OK018167 |
| *BRCA2* | c.6325G>A; p.Val2109Ile | OK018168 |
| *BRCA2* | c.7052C>G; p.Ala2351Gly | OK018169 |
| *BRCA2* | c.7102T>G; p.Leu2368Val | OK018170 |
| *BRCA2* | c.7426_7427delinsCC; p.Glu2476Pro | OK018171 |
| *BRCA2* | c.8009C>T; p.Ser2670Leu | MG494329 |
| *BRCA2* | c.8023A>G; p.Met2676_Ile2778del | KJ625195 |
| *BRCA2* | c.8162T>A; p.Leu2721His | OK018172 |
| *BRCA2* | c.9538C>T; p.Leu3180Phe | OK018173 |
